# Transcriptome Characterization of *Cymbidium sinense *'Dharma' Using 454 Pyrosequencing and Its Application in the Identification of Genes Associated with Leaf Color Variation

**DOI:** 10.1371/journal.pone.0128592

**Published:** 2015-06-04

**Authors:** Genfa Zhu, Fengxi Yang, Shanshan Shi, Dongmei Li, Zhen Wang, Hailin Liu, Dan Huang, Caiyun Wang

**Affiliations:** 1 Guangdong Key Laboratory of Ornamental Plant Germplasm Innovation and Utilization, Environmental Horticulture Research Institute, Guangdong Academy of Agricultural Sciences, Guangzhou, 510640, P. R. China; 2 College of Horticulture & Forestry Sciences, Huazhong Agricultural University, Wuhan, Hubei, 430070, P. R. China; ISA, PORTUGAL

## Abstract

The highly variable leaf color of *Cymbidium sinense* significantly improves its horticultural and economic value, and makes it highly desirable in the flower markets in China and Southeast Asia. However, little is understood about the molecular mechanism underlying leaf-color variations. In this study, we found the content of photosynthetic pigments, especially chlorophyll degradation metabolite in the leaf-color mutants is distinguished significantly from that in the wild type of *Cymbidium sinense *'Dharma'. To further determine the candidate genes controlling leaf-color variations, we first sequenced the global transcriptome using 454 pyrosequencing. More than 0.7 million expressed sequence tags (ESTs) with an average read length of 445.9 bp were generated and assembled into 103,295 isotigs representing 68,460 genes. Of these isotigs, 43,433 were significantly aligned to known proteins in the public database, of which 29,299 could be categorized into 42 functional groups in the gene ontology system, 10,079 classified into 23 functional classifications in the clusters of orthologous groups system, and 23,092 assigned to 139 clusters of specific metabolic pathways in the Kyoto Encyclopedia of Genes and Genomes. Among these annotations, 95 isotigs were designated as involved in chlorophyll metabolism. On this basis, we identified 16 key enzyme-encoding genes in the chlorophyll metabolism pathway, the full length cDNAs and expressions of which were further confirmed. Expression pattern indicated that the key enzyme-encoding genes for chlorophyll degradation were more highly expressed in the leaf color mutants, as was consistent with their lower chlorophyll contents. This study is the first to supply an informative 454 EST dataset for *Cymbidium sinense *'Dharma' and to identify original leaf color-associated genes, which provide important resources to facilitate gene discovery for molecular breeding, marketable trait discovery, and investigating various biological process in this species.

## Introduction

*Cymbidium* is an economically important genus of flowering orchids cultivated in China, Japan, Korea, and Southeast Asia [1, 2]. Due to its beautiful, fragrant flowers and elegant, upright leaves, *Cymbidium* is very popular in China and is highly desirable in traditional flower markets. The floral characteristics of *Cymbidium* have previously attracted the most horticultural attention [3, 4], but leaf variations such as striped leaves, transparent leaves, yellow leaves, and spotted leaves have recently become of interest [5–7]. Leaf color variation significantly improves the horticultural and economic value of *Cymbidium* and has therefore become one of the main focuses of its cultivation and breeding [8]. Among the diverse *Cymbidium* cultivars, 'Dharma' is a typical *Cymbidium sinense* cultivar exhibiting highly variable leaf color and highly improved economic value [9]. More than 30 leaf color mutations have been found in *Cymbidium sinense* 'Dharma' to date [9], including claw, spot, and crane mutations, which makes the cultivar an optimal model for studying the leaf variation of *Cymbidium* orchids.

However, due to the polyploid genomes and long juvenile phases of the genus, functional genomic studies and gene discovery of valuable horticultural traits are greatly limited in *Cymbidium* orchids, make it singularly challenging to study and improve using classical genetic approaches [10, 11]. Molecular technology for character improvement in *Cymbidium* is currently scarce, seriously hindering the development of the orchid industry [12]. In recent years, the rapid expansion of next-generation sequencing (NGS) technologies has provided powerful tools for high-throughput sequence determination [13–15]. Various NGS-based RNA sequencing techniques have made sequencing cheaper and faster, allowing for the easy discovery of novel genes by obtaining massive amounts of sequence data with enormous depth and coverage [15]. Among these techniques, massively parallel 454 pyrosequencing, which produces long, credible reads, is particularly useful for determining gene expression, especially in nonmodel plants for which genomic sequence data are unavailable [16, 17]. This technology can be used to deeply explore the nature and complexity of a given transcriptional universe. A number of very recent studies have highlighted the utility of cDNA sequencing in discovering candidate genes for floral development, floral scent production, and flowering time in several orchid genus such as *Phalaenopsis*, *Dendrobium*, *Vanda* and *Oncidium* orchids, for which no genomic sequences exist [18–22]. However, the molecular mechanism underlying leaf color variation has received little previous attention. Especially for *Cymbidium* orchids, a comprehensive description of the full complement of expressed genes remains unavailable, and the candidate genes related to leaf color variation are poorly understood [23–25].

In this study, we examined the control of chlorophyll content in the mutant leaves, and found the content of chlorophyll synthesis precursors decreased slightly and the level of chlorophyll degradation metabolite increased significantly compared to that in the wild type, which further resulted in apparent reduction of photosynthetic pigments in the mutant leaves. To determine functionally characterized candidate genes that may be directly associated with the chlorotic leaves in *Cymbidium sinense* and obtain an overview of the gene expression, we sequenced the transcriptome of *Cymbidium sinense* 'Dharma', a typical *Cymbidium sinense* cultivar well-known for its leaf color variations [9], and identified 95 isotigs involved in chlorophyll metabolism.

We further determine the suitability of our *de novo* assembly and annotation of the expressed genes, and identified 16 key enzyme-encoding genes in the chlorophyll metabolism pathway. Our real-time PCR results validated that the expression levels of two key enzyme-encoding genes for chlorophyll degradation were higher in yellow-colored leaves, whereas four of the key enzyme-encoding genes for chlorophyll biosynthesis showed almost the same expression than that of the wild type. This result was consistent with the higher level of chlorophyll degradation metabolite and lower chlorophyll contents measured in the mutant leaves, and further revealed that the leaf color variation is probably owing to over degradation of chlorophyll rather than biosynthesis deficiency.

To our knowledge, this study represents the first characterization of the *Cymbidium sinense* 'Dharma' transcriptome using massively parallel 454 pyrosequencing. This informative EST dataset provides an important resource for marketable trait discovery and investigating various biological processes in *Cymbidium*. Additionally, our study on leaf variation will provide valuable data and practical guidance for improving horticultural traits and facilitating molecular breeding in *Cymbidium*.

## Materials and Methods

### Plant materials and growth conditions

Wild-type plants and leaf-color mutants of *Cymbidium sinense* 'Dharma' used in this study were collected from the cultivation base of floricultural research institute, Guangdong Academy of Agricultural Sciences, China. All of the plants were grown and maintained in pots in a greenhouse at day/night temperatures of 26/23°C under a 16-h light /8-h dark photoperiod.

### Biochemical measurements

For Chlorophyll content measurement, six-month old leaves of wild type and the mutant grown under identical conditions were collected and Chl (a,b) were extracted in 90% acetone and determined spectrophotometrically by the method of Lorenzen [26]. For the contents of chlorophyll intermediate products, leaves were homogenized in nine volume of 0.01 M Phosphate Buffered Saline (PBS), and mixed on the ice and centrifuged (30 min at 2,500g). The supernatants was assayed by a ELISA kit (HengYuan Biological Technology Co.,Ltd, Shanghai, China) separately. Data were analyzed by SPSS software.

### cDNA preparation and 454 sequencing

**Fig 1:**
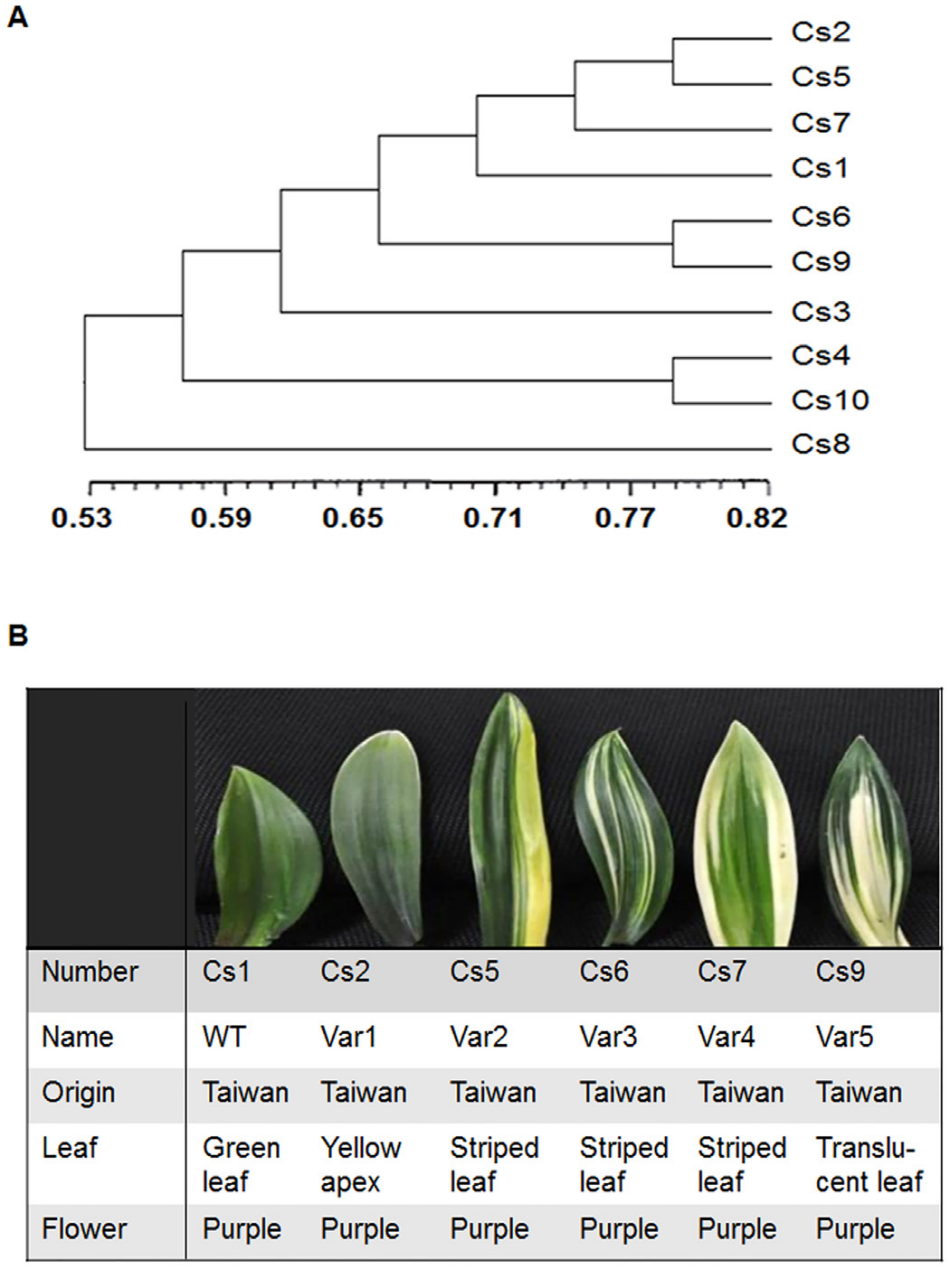
Morphologic and Phylogenetic analysis of different-colored mutants of *Cymbidium sinense* 'Dharma.' (A) The UPGMA cluster of ten leaf-color mutants of *Cymbidium sinense* 'Dharma' based on AFLP markers; (B) Morphologic characteristics of six leaf-color mutants of *Cymbidium sinense* 'Dharma' studied in this work.

The cDNA libraries were prepared from different tissues of *Cymbidium sinense* 'Dharma' as shown in S1 Fig, including roots, leaves, pseudobulbs and flowers. The leaf samples were collected from six-month old (young leaf, YL) and three-year old (old leaf, OL) leaves of wild-type plants, as well as the six-month old leaves of various leaf-color mutants (Fig 1,Var1-5). Total RNA was extracted from approximately 0.5 g of each tissue using the Trizol reagent (Life Technologies). Individual mRNAs were purified with an Oligotex mRNA Midi Kit (QIAGEN). First-strand cDNA was synthesized using SuperScript II (Life Technologies) with oligo(dT)15-VNN as the template-primer, according to the manufacturer’s protocol. Double-stranded cDNA was synthesized using a SuperScript double-stranded cDNA synthesis kit (Life Technologies) with the provided primers in a 100 mμl reaction. The double-stranded cDNA was sequenced using a 454 GS-FLX instrument (Roche Applied Science) and the sequencing was performed at Shanghai Majorbio Bio-pharm Biotechnology Co., Ltd. (Shanghai, China).

### 454 EST assembly and annotation

Prior to assembly, the resulting 454 raw reads that failed to pass the GS FLX pyrosequencing filter (eg. reads with weak signal or low quality) were discarded to yield a high-quality (HQ) data set (>99.5% accuracy of single-base reads). Primer and adapter sequences were trimmed from the HQ dataset, non-coding RNA (such as rRNA,tRNA, and miRNA) and sequences shorter than 50 bp were also removed. The remaining data were assembled into unique sequences using Trinity (http://trinityrnaseq.sourceforge.net/analysis/extract_proteins_from_trinity_transcripts.html) with an optimized k-mer length of 25.

Open Reading Frame (ORF) survey were carried out using TransDecoder software to find protein-coding sequences[27], which further were searched against the NCBI non-redundant protein (Nr) database (http://www.ncbi.nlm.nih.gov) the Swiss Protprotein database (http://www.expasy.ch/sprot), and the Uniprot database (http://www.uniprot.org/) using the BLASTP algorithm with an E-value cut-off of 10^-5^. The remaining sequences were searched against these public databases using the BLASTX algorithm with an E-value cut-off of 10^–5^.

The BLAST2GO V. 2.4.4 was used to obtain the Gene Ontology (GO) classification (http://wego.genomics.org.cn/cgi-bin/wego/index.pl) of unique transcripts [28]. The assembled sequences were also aligned to the COG database to predict and classify functions [29]. Metabolic pathway assignments was performed based on the pathways of *Arabidopsis thaliana* and *Oryza sativa* in the Kyoto Encyclopedia of Genes and Genomes (KEGG) mapping project (http://www.genome.ad.jp/kegg/kegg2.html) [30]. Enzyme commission (EC) numbers were assigned to unique sequences that had BLASTX scores with an E-value cut-off of 10^-5^ as determined by searching the KEGG database.

### Sequence Alignment and Phylogenetic Analysis

Full-length amino acid sequences were aligned by the Clustal X 1.83 program. The sequence alignment was further adjusted manually using BioEdit software (http://www.mbio.ncsu.edu/bioedit/bioedit.html). The amino acid substitution model was calculated by the Model Generator v0.84 and the optimal model of “JTT+G” was selected. Phylogenetic relationships were reconstructed using a maximum-likelihood (ML) method in PHYML software with JTT amino acid substitution model.

### Northern blot analysis

Total RNA was extracted from plant tissues using Trizolreagent (Life Technologies), separated on 1.5% formaldehyde-MOPS agarose gels and blotted onto N^+^ membranes (Millipore Corporation, http://www.millipore.com). Hybridization was performed at 68°C in Perfect HybTMPlus Buffer (Sigma-Aldrich). Probes were labeled with 32^P^-dATP using the Klenow fragment (Takara).

### Real-time quantitative RT-PCR (qRT-PCR) analysis

For expression levels of the genes related to chlorophyll metabolism pathway, six-month old leaves of wild-type and five leaf color-mutants were harvested for RNA extraction by the use of RNAiso Plus kit (Takara). 1μg of total RNA was quantified using a NanoDrop 2000 spectrophotometer (Thermo Scientific) for reverse-transcription (Vilnius, Lithuania). Relative transcripts levels were determined using the iCycler IQ Real-time PCR Detection System (Bio-Rad, USA) according to the manual QuantiTect SYBR Green PCR kit and analyzed by icycler real-time detection system software (version 5.0). Expression levels were normalized using the expression level of the constitutive actin gene. A relative quantitative method (DDCt) was used to evaluate the quantitative variation. All quantifications were made in duplicate on RNA samples obtained from three independent experiments. The primers used for real-time PCR amplifications are listed in Supplemental S1 Table.

## Results and Discussion

### The morphology and Phylogenetic analysis of *Cymbidium sinense* 'Dharma'

In the process of cultivation and domestication of orchid species, color mutation in the leaves occurred at very high frequencies,resulting in different-colored mutant. The most representative cultivar, *Cymbidium sinense* 'Dharma', has generated more than 30 types of leaf variation by far, which makes it an optimal model to study leaf variation in *Cymbidium* family.

To illustrate the mechanism underlying leaf color variation, we collected ten different-colored mutants of *Cymbidium sinense* 'Dharma', and examined the associations of the genetic polymorphism with their morphological traits by amplified fragment length polymorphism analysis (AFLP). Consequently, 841 bands from 9 primer combinations were obtained, 644 of which (76.6%) were polymorphic ones. AFLP analysis showed genetic similarity coefficients among them were ranged from 0.6164 to 0.8245, with an average value at 0.7256 (S2 Table). Using UPGMA cluster analysis, these ten cultivars can be divided into two clusters, which basically agreed with those of morphological taxonomic study and also are related well with the origin of them (Fig 1A and S3 Table). On this basis, five mutants that cluster together were selected for further analysis (Fig 1B).

### Decreased content of photosynthetic pigments in the mutant leaves

**Fig 2:**
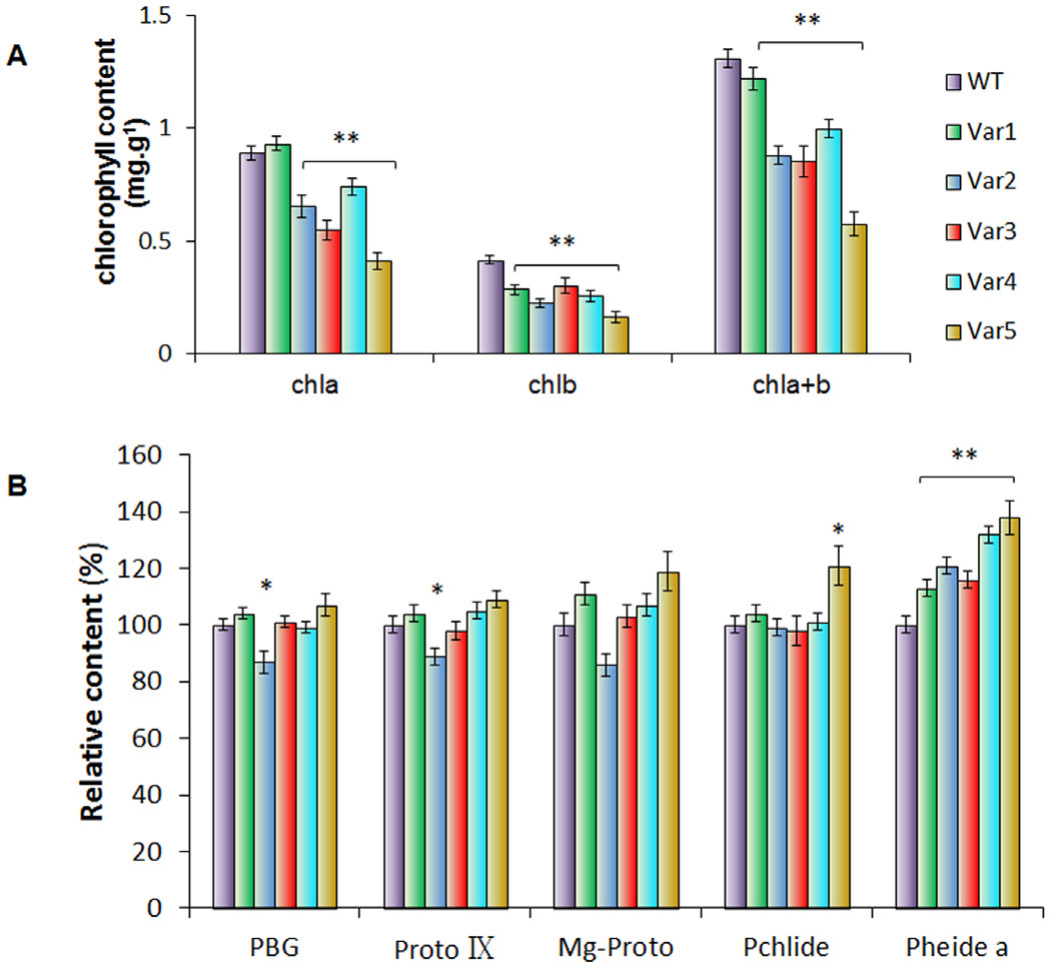
The variations in the contents of photosynthetic pigments between the wild type and leaf-color mutants. (A) Chlorophyll content in wild-type and color-mutant leaves of *Cymbidium sinense* 'Dharma'(mg g^-1^ FW); (B) The relative contents of chlorophyll synthesis precursors PBG, Proto IX, Mg-Proto, and Pchlide, and chlorophyll degradation metabolite Pheide a (Take each intermediate product content in the wild type as 100%, comparing it with its mutant correspondent). Values are means ±standard deviation (n > 10). Mean-Whitney U-test significant at *P<0.05 between the mutants and the wild type control.

The mutant showed white-yellow band in the leaf from the seedling stage to the maturity that cannot turn to green with environmental stimulations. Meanwhile, chlorophyll levels in the mutant leaves differed significantly from that in the wild-type leaves, which showed approximately 60% to 17% decrease of the chlorophyll a content, and 73% to 32% decrease of the chlorophyll b content, respectively, compared with the wild-type (Fig 2A). Moreover, detailed analysis showed that the content of chlorophyll synthesis precursors porphobilinogen (PBG), protoporphyrin IX (Proto IX), magnesium protoporphyrin (Mg-Proto), and protochlorophyllide (Pchlide) decreased slightly in most of the mutant leaves. On the contrary, the level of chlorophyll degradation metabolite Pheophorbide acid a (Pheide a) increased apparently, and the changing was consistent with the chlorotic level of the mutant leaves. As shown in Fig 2B, the content of photosynthetic pigments PBG, Proto IX, and Mg-Proto in the mutant leaves decreased from 0–13%, 0–11%, and 0–14%, respectively. Contratrarirly, the level of Pheide a increased from 13%-38% in severe chlorotic leaf of the mutant compared with that of wild type. This result implied that the yellow and green striped leaf spots in *Cymbidium* 'Dharma' were possibly due to over degradation of Chlorophyll rather than biosynthesis deficiency.

### Generation and assembly of 454-pyrosequenced expressed sequence tags (ESTs)

**Fig 3:**
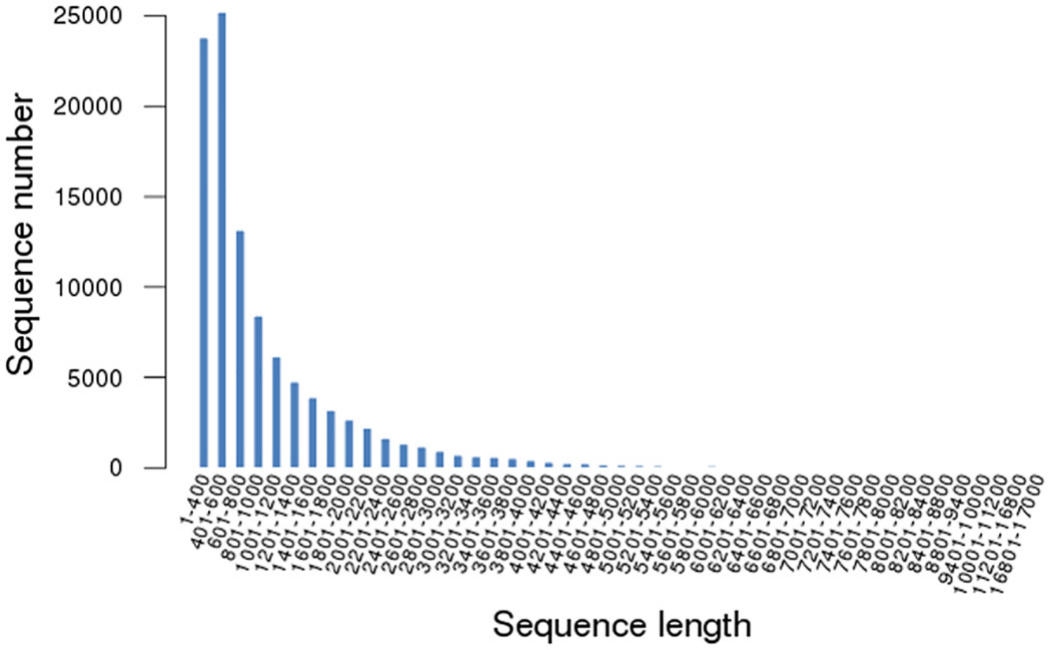
The size distribution of assembled isotigs. The x-axis represents the sequence length in base pairs. The y-axis represents the isotigs number.

To illustrate the potential mechanism of leaf color variation and find candidate genes involved in this process, we firstly generated a cDNA library for 454 GS-FLX sequencing from an equal mixture of RNAs isolated from different organs, and obtained an overview of the expressed genes in *Cymbidium sinense* 'Dharma' (S1 Fig). A total of 705,630 raw reads with an average length of 456.7 bp were generated from one pyrosequencing run. After removing the adaptor reads, repeat sequences, and low-quality reads, 702,015 high-quality reads, totaling 313 Mb with an average length of 445.9 bp, were obtained for further analysis. *De novo* assembly of these clean reads using the Trinity program generated 103,295 isotigs with 104,336,567 total residues, representing 68,460 genes (Table 1). Isotig length averaged 1,010 bp and ranged from 306 to 16,856 bp. The size distribution of the assembled isotigs is shown in Fig 3. Although most of the isotigs were between 400 and 600 bp in length, a substantial number of large isotigs were obtained. A total of 8,573 isotigs were longer than 2,000 bp, and 29,206 isotigs were longer than 1,000 bp. These data provided comprehensive gene expression information to facilitate the investigation of *Cymbidium* genetics.

**Table 1:** Summary of sequencing and *de novo* assembling of the transcriptome in *Cymbidium sinense* 'Dharma'.

Summary of *C*. *sinense* 'Dharma' EST data	
Total number of raw reads	705,630
Total number of clean reads	702,015
Total clean nucleotides (nt)	313,031,972 bp
Average read length	445.9 bp
Total genes	68,460
Total isotigs	103,295
Total residues	104,336,567 bp
Smallest isotig	306 bp
Largest isotig	16,856 bp
Average length	1010.08 bp

### Annotation of the novel transcripts

**Fig 4:**
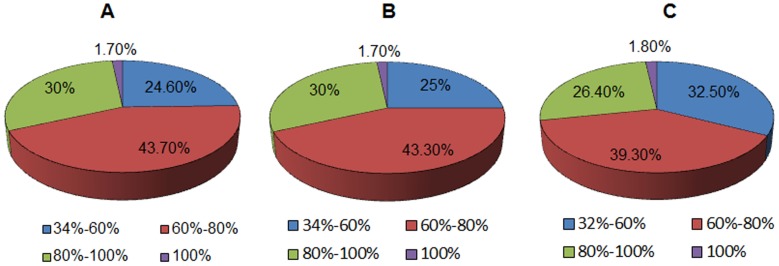
Characteristics of homology search of the isotigs. (A) Similarity distribution of the best Blast hits in Nr database; (B) Similarity distribution of the best Blast hits in Uniprot database; (C) Similarity distribution of the best Blast hits in Swissport database.

To validate the effectiveness and suitability of our *de novo* assembly, all of the assembled sequences were first surveyed by open reading frame (ORF) using TransDecoder software (as described in “Materials and Methods”). This analysis produced 46,210 protein-coding isotigs containing at least an ORF, and the remaining parts were considered as noncoding sequences. Both the putative protein-coding and noncoding transcripts were further searched against the NCBI nonredundant (NR) protein database, as well as the UniProt and SwissProt databases, using the BLASTp and BLASTX algorithms, respectively. A total of 43,433 significant matches were obtained in the NR database (Table 2), and the similarity distribution showed that 31.7% of these alignments had a similarity higher than 80%, 43.7% between 60% and 80%, and 24.6% lower than 60% (Fig 4A). UniProt alignment indicated 43,542 matches and showed a similarity distribution pattern consistent with that observed in the NR database, as shown in Fig 4B. Additionally, nearly 30% (30,629) of the predicted isotigs matched to sequences in the SwissProt database, and the similarity distribution pattern was comparable to that of the other databases, with 26.4% of the sequences having a similarity higher than 80% and over 70% of the hits having a similarity from 32% to 80% (Fig 4C).

**Table 2:** Summary of the blast hits against the known protein database.

	Number	Percent (%)
Total	103,295	
ORF	46,210	44.74
Nr	43,433	42.05
Uniprot	43,542	42.15
Swissprot	30,629	29.65
GO	29,299	28.36
String	11,157	10.80
COG	6,913	6.69
KOG	7,772	7.52
KEGG	12,852	12.44

As “non-BLASTable” sequences have been reported in all studied plant transcriptomes, with proportions varying from 13% to 80% depending on species and sequencing depth [31, 32] (e.g. primarily 5’and 3’UTR fractions, lineage-specific genes and fast-evolving genes existing in the dataset), it is reasonable that nearly half of the assembled sequences did not receive putative functional identifications in our annotation.

### Gene ontology (GO) and clusters of orthologous groups (COG) classification

**Fig 5:**
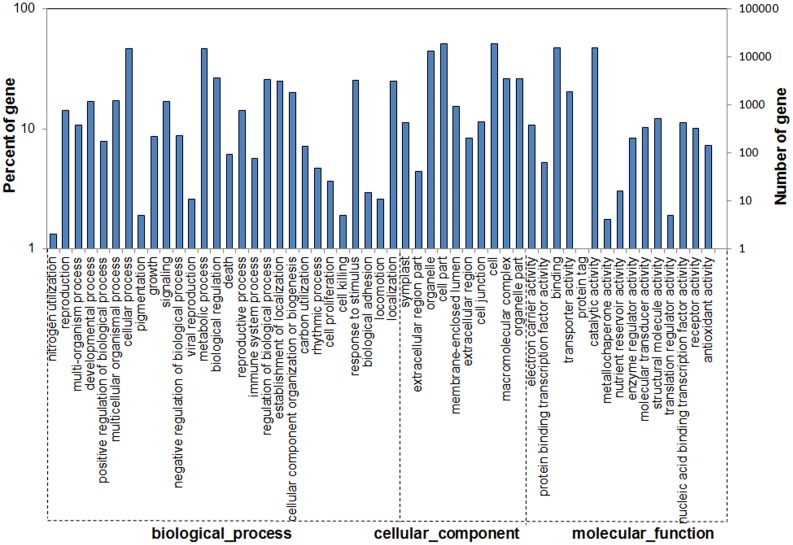
GO classification of unigenes of assembled isotigs.

We used the GO system to classify the functions of the predicted isotigs of *Cymbidium* 'Dharma'. A total of 29,299 isotigs were categorized into 42 functional groups. "Metabolic process" and "cellular process" were the most highly represented groups in the biological process category, suggesting that our study may allow for the identification of novel genes involved in secondary metabolite synthesis pathways. "Cell " and "cell part" terms were dominant among the cellular component categories. Among the molecular function categories, "catalytic activity" had the most number of isotigs, followed by "binding". However, only a few genes were identified for "nitrogen utilization" (2), "pigmentation" (5), "viral reproduction" (11), "cell killing" (5), "locomotion" (11), and" biological adhesion" (15) in the molecular function category, and a limited number were found for "metallochaperone activity" (4), "translation regulator activity" (5), "translation regulator activity" (5), and "protein tags" (1) in the cellular component category (Fig 5).

To generate a focused view of the GO categories, further functional classification of all isotigs was performed using a set of plant-specific GO slims. "Plastid", "cell", "biosynthetic process ", and "metabolic process" were the most highly represented groups, followed closely by "cellular process", "mitochondrion", "protein modification process", "transport", and "cytoplasmic membrane–bounded". "Stress response", "signal transduction", "cell cycle", and "photosynthesis" were also represented (S4 Table). These trends were consistent with overall biological activity during the plant developmental periods.

**Fig 6:**
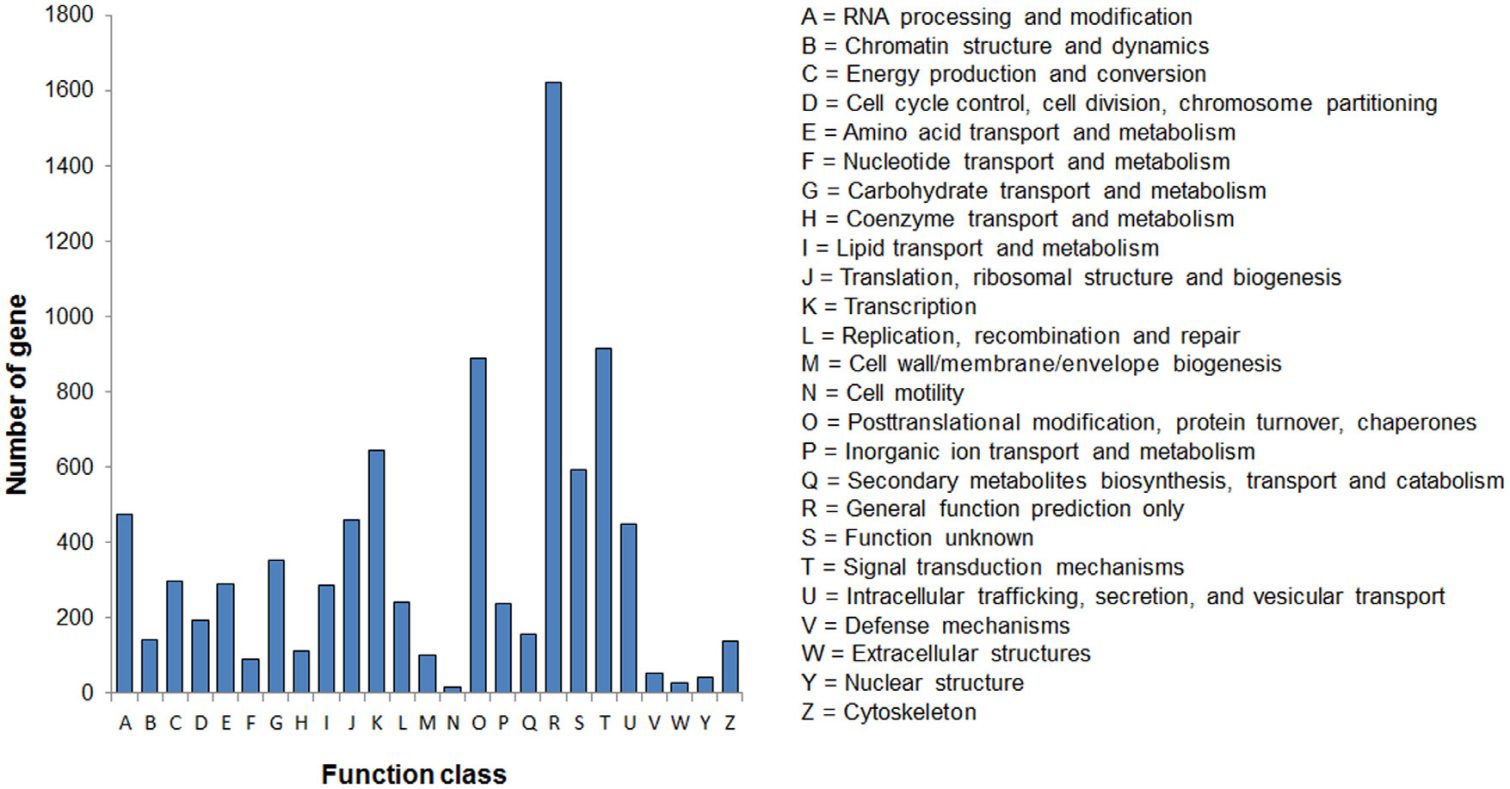
KOG function classification of assembled isotigs.

We further classified the isotigs using the KOG system. Based on sequence homologies, 10,079 isotigs were classified into 25 KOG functional classifications. Among these classifications, the "general function prediction" cluster represented the largest group, followed by "posttranslational modification", "signal transduction" "Transcription", and "Cell motility" was the smallest group, with only eight identified candidates (Fig 6).

### Gene pathway assignments by the Kyoto Encyclopedia of Genes and Genomes (KEGG)

We mapped the assembled isotigs to the reference canonical pathways in the Kyoto Encyclopedia of Genes and Genomes (KEGG), including metabolism, genetic information processing, environmental information processing, cellular processes, and organism systems (http://www.genome.jp/kegg/pathway.html). In total, 21,633 sequences were mapped to 293 KEGG pathways, representing 31.6% of the assembled isotigs (S5 Table). Among these isotigs, the most (12,335) were related to metabolism, while 3,687 corresponded to genetic information processing, 1,069 mapped to environmental information processing, 1,885 were associated with cellular processes, and 2,657 belonged to organism systems (Table 3). These pathways provide valuable resources for investigating specific metabolic processes during *Cymbidium* 'Dharma' research. The presence of genes for these essential cellular processes suggests that these sequences cover most of the *Cymbidium* 'Dharma' transcriptome, which could further facilitate the identification of gene sets involved in a broad range of biological processes.

**Table 3:** Functional categories of assembled isotigs in KEGG pathways.

	Sub pathways of KEGG	Gene number
Metabolism		12335
	Global map	5113
	Carbohydrate metabolism	1582
	Energy metabolism	837
	Lipid metabolism	816
	Nucleotide metabolism	761
	Amino acid metabolism	976
	Metabolism of other amino acids	344
	Glycan biosynthesis and metabolism	479
	Metabolism of cofactors and vitamins	461
	Metabolism of terpenoids and polyketides	294
	Biosynthesis of other secondary metabolites	343
	Xenobiotics biodegradation and metabolism	329
Genetic Information Processing		3687
	Transcription	472
	Folding, sorting and degradation	1161
	Replication and repair	686
	Translation	1368
Environmental Information Processing		1069
	Membrane transport	114
	Signal transduction	954
	Signaling molecules and interaction	1
Cellular Processes		1885
	Transport and catabolism	643
	Cell motility	111
	Cell growth and death	929
	Cell communication	202
Organismal Systems		2317
	Immune system	445
	Endocrine system	511
	Circulatory system	95
	Digestive system	268
	Excretory system	156
	Nervous system	672
	Sensory system	100
	Development	70
	Environmental adaptation	340

### Identifying putative genes related to leaf color variations

**Fig 7:**
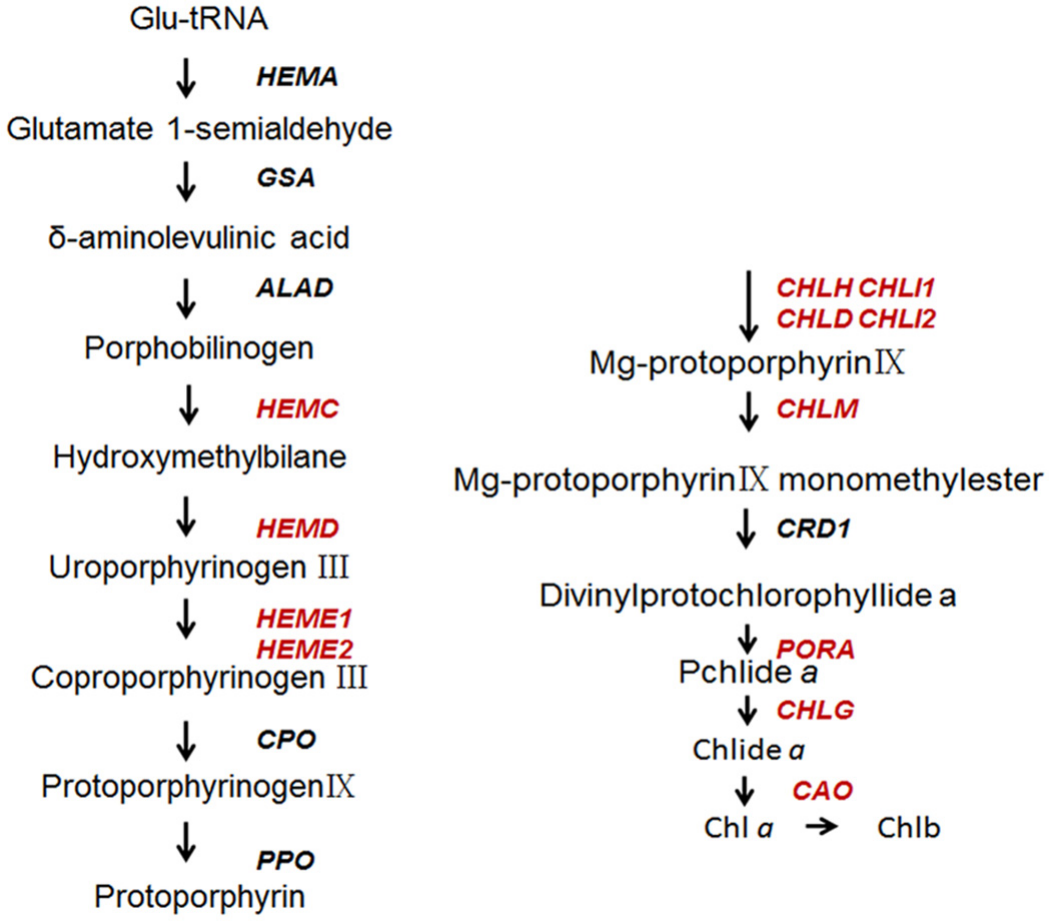
Assembled isotigs involved in the chlorophyll biosynthesis pathway pathway of *Cymbidium sinense* 'Dharma'. Abbreviation: HEMC, hydroxymethylbilane synthase; HEMD, uroporphyrinogen-III synthase; HEME, uroporphyrinogen decarboxylase; CHLH, magnesium chelatase; CHLD, magnesium chelatase subunit ChlD; CHLI, magnesium chelatase subunit ChlI; CHLM, magnesium-protoporphyrin O-methyltransferase; PORA, protochlorophyllide oxidoreductase A; CHLG, Chlorophyll synthase; CAO, chlorophyllide *a* oxygenase.

Our result showed that the chlorophyll contents decreased significantly in severe chlorotic leaf of the mutant compared with that of wild type. We therefor concentrated on the orthologs of genes that are known to be involved in chlorophyll metabolism, and identified 95 isotigs as putative chlorophyll-related genes (S6 Table). According to the previous studies in *Arabidopsis*, chlorophyll synthesis begins with glutamyl-tRNA (Glu-tRNA), is catalyzed by 16 enzymes, and finally forms chlorophyll b through 16 key regulatory steps [33]. Changes at any step will affect the abundance of chlorophyll pigment, causing leaf color variation [33–35]. Homologous sequences for these key enzyme-coding genes were found in our EST dataset (highlighted in red in Fig 7 and listed in Table 4).

**Table 4:** Putative key enzyme-encoding genes in chlorophyll metabolism pathway.

	Gene ID	Homologous gene	gene Nr-ID	Similarity Nr_top (%)
hlorophyll biosynthesis	comp4803_c0 comp7390_c0 comp130_c0 comp8839_c0 comp3717_c0 comp5844_c0 comp4433_c0 comp10192_c0 comp7316_c0 comp2796_c0 comp434_c0 comp3345_c0 comp8089_c0 comp1664_c0 comp255_c0	CHLG PORA PPOX HEME1 HEME2 HEMD HEMC CHLD CHLH CHLI CHLM CAO	AEI83422.1 CBW30171.1 XP_002264042.1 XP_002263321.1 XP_002515173.1 CBI20191.3 XP_002274385.2 XP_003603007.1 CBI18797.3 XP_002513534.1 XP_003535922.1 XP_002316838.1 XP_002280872.1 XP_002306638.1 XP_002324928.1	90 77 89 80 84 83 92 83 91 90 92 85 83 89 77
Chlorophyll metabolism	comp1087_c0 comp3334_c0 comp3012_c0 comp6651_c0 comp1152_c1	PAO CLH2 RCCR NOL	XP_002274210.1XP_002533392.1 XP_002517075.1CAJ87104.1 XP_002266979.1	81 71 76 71 96

**Fig 8:**
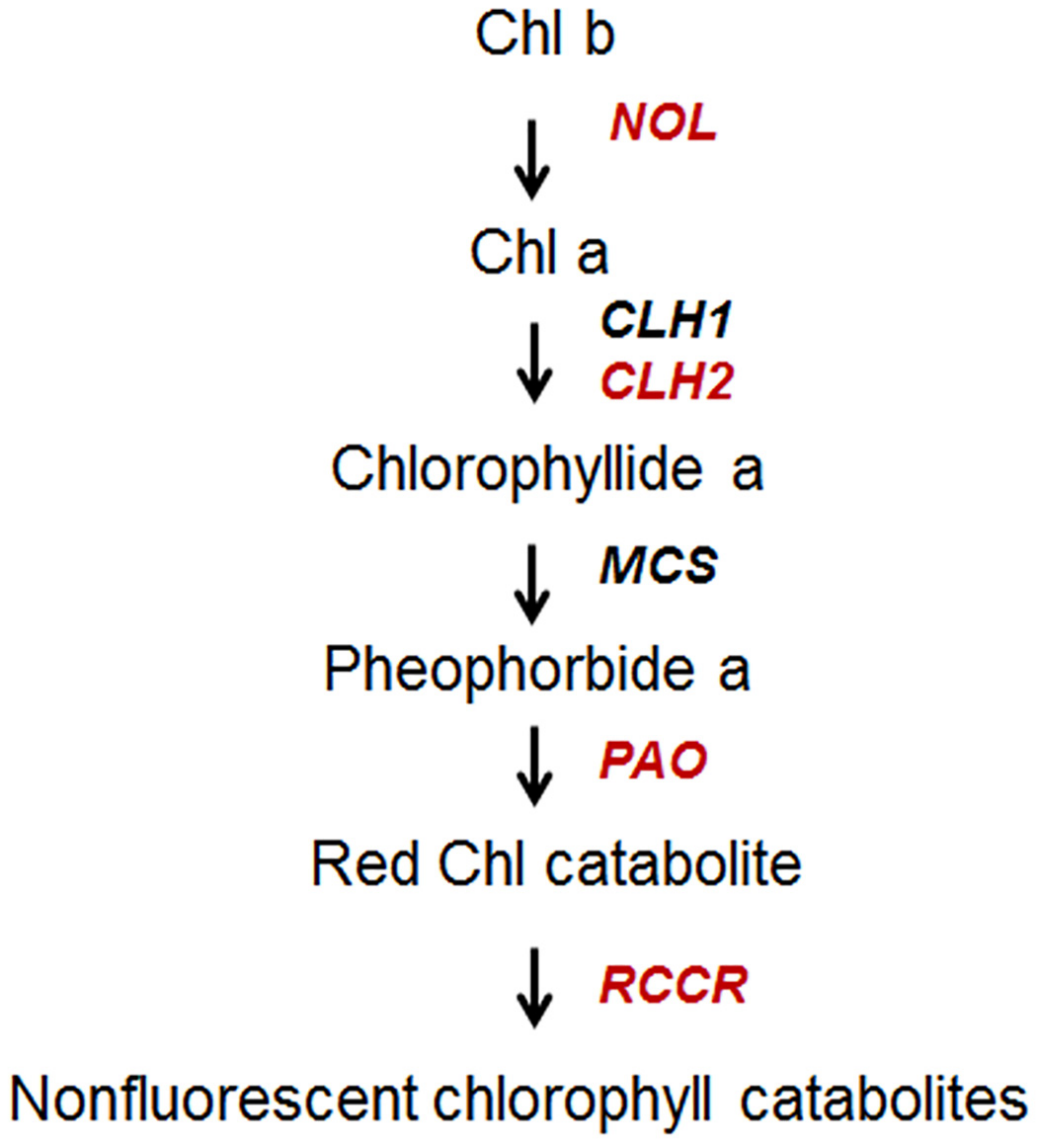
Assembled isotigs involved in the chlorophyll degradation pathway pathway of *Cymbidium sinense* 'Dharma'. Abbreviation: NOL, chlorophyll b reductase; CLH, chlorophyllase; PAO, pheophorbide a oxygenase, RCCR, red Chl catabolite reductase.

Besides, candidate genes associated with chlorophyll degradation were also evaluated in our sequencing data. According to the proposed pathway of chlorophyll catabolism in *Arabi*dopsis [35,36], we identified homologous sequences of genes encoding key enzymes for chlorophyll degradation, including chlorophyll-chlorophyllido hydrolases (CLHs), pheophorbide an oxygenase (PaO), red Chl catabolite reductase (RCCR), and chlorophyll b reductase (NOL) (highlighted in red in Fig 8 and listed in Table 4).

To validate the discovery methods, we cloned the full lengths of the ORFs by rapid amplification of the 5′ cDNA end (5′RACE). Full-length cDNAs were obtained for eight identified genes, *CHLG*, *PORA*, *HEME2*, *HEMD*, *HEMC*, *CAO*, *CLH2*, and *RCCR*, and all were identical to the RNA-seq data (S7 Table). We randomly chose the homologous sequences of chlorophyll synthase, CHLG (comp4803_c0), and chlorophyllase, CLH2 (comp3012_c0), for further analysis. Our result showed that their ORF were of parallel sizes to their homologs in *Arabidopsis* (shown in S2 and S3 Figs, respectively). Homologous alignment also indicated comparative high similarity with other species (shown in S4 and S5 Figs, respectively), which confirmed that our 454 EST dataset could effectively facilitate gene identification. This systems bioinformatics survey, combined with molecular biology analysis, is also a feasible approach to elucidate the scent of various biological processes and identify the relevant genes.

### Validation and expression analysis of key enzyme genes

**Fig 9:**
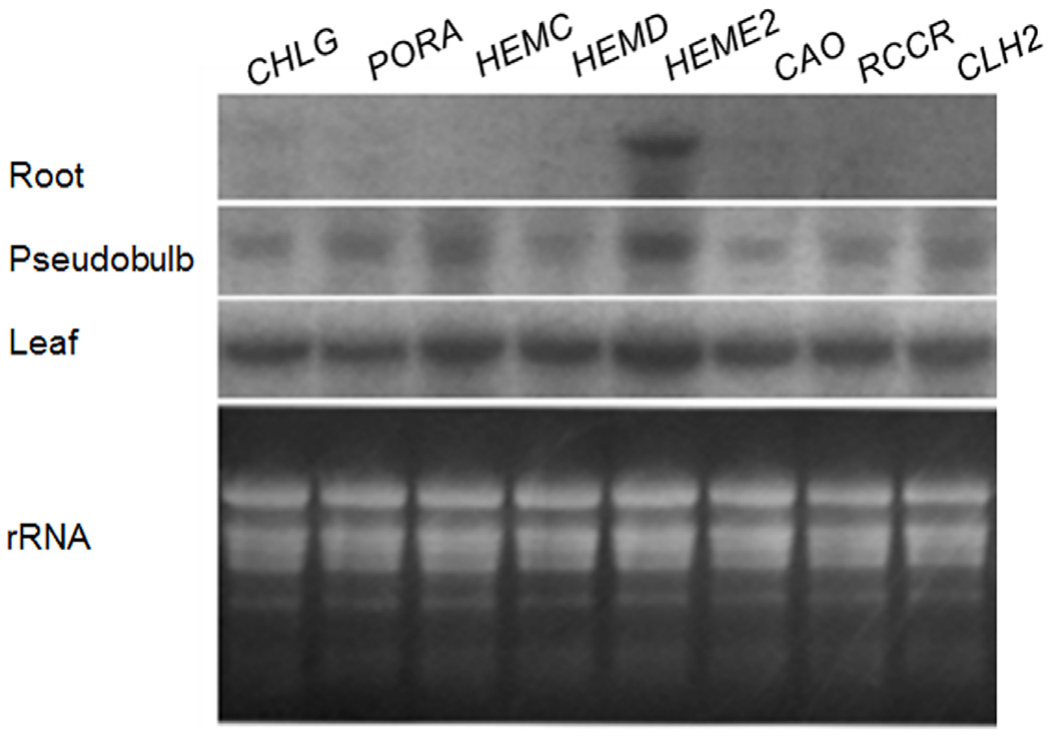
Northern blotting analysis of the expression of identified genes in different tissues of *Cymbidium sinense* 'Dharma'. Each lane contained 15 g total RNA isolated from roots, pseudobulbs and six-month old leaves, rRNA served as a loading control (bottom of the panel).

The expressions of the eight identified homologous genes encoding key enzymes in the chlorophyll biosynthetic and catabolic pathways were further confirmed by RNA blot hybridization. Our results showed that all six of the candidate genes involved in chlorophyll biosynthesis were highly expressed in the leaves and pseudobulbs but rarely expressed in the roots, as was consistent with the sole deposition of chlorophyll in the leaves (Fig 9). Likewise, the homologs of chlorophyll catabolism genes *CHL2* and *RCCR* were also expressed to a greater extent in the leaves, in agreement with the pattern of chlorophyll distribution.

**Fig 10:**
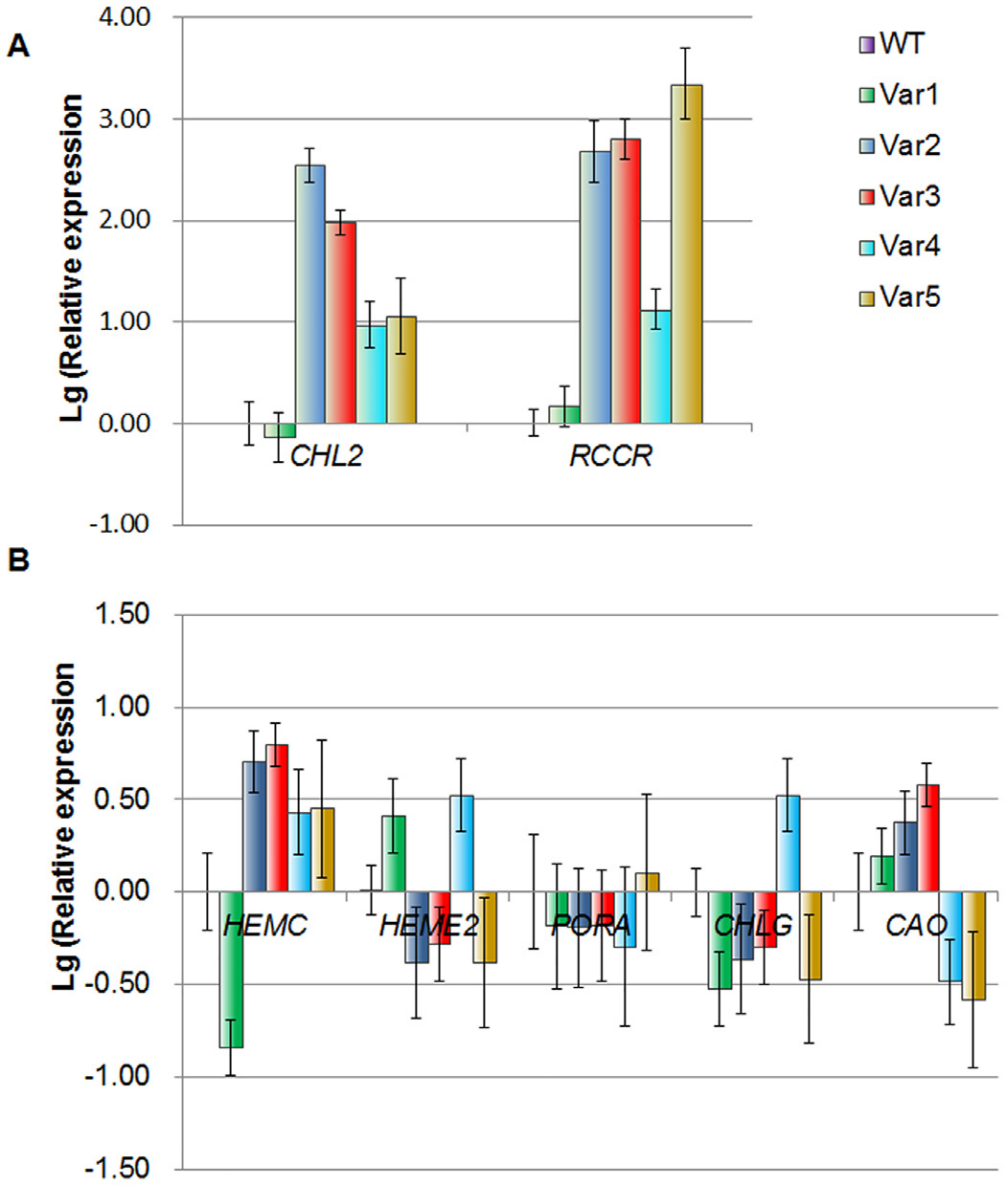
Gene expression analysis. The qRT-PCR analysis of the expressions of two enzyme-encoding genes in chl degradation pathway (A) and five key enzyme-encoding genes in chl biosynthesis pathway (B). The y-axis indicates fold change in expression among the samples. The Lg(Relative Quantitation) of the genes in the wild-type leaves was calibrated as zero. RNA was extracted from six-month-old normal growth leaves. *ACT* gene served as the internal control. Error bars indicate the standard deviation of the mean (SD) (n = 3). Three biological replicates were analyzed, with similar results.

Quantitative PCR analysis revealed that the expression level of *RCCR* in the leaves of yellow-leaf mutants was one- to nine-fold higher, and those of *CHL2* approximately one- to five-fold higher, than in the leaves of wild-type 'Dharma' under normal growth conditions and the changing was consistent with the chlorotic level of the mutant leaves (Fig 10A). By contrarily, the transcript abundances of the key genes for chlorophyll biosynthesis were nearly the same for both yellow-leaf mutants and wild-type plants. As shown in Fig 10B, the differences in expression are no more than 1.5 folds and did not correlate well with their chlorotic levels. This result indicates that the chlorophyll degradation genes were expressed in the yellow-leaf mutants at a higher level, as was consistent with their lower chlorophyll contents and higher chlorophyll degradation.

## Conclusion

In this study, we examined the differences between the normal and color-mutant leaves of *Cymbidium sinense* cultivar 'Dharma', and found a notable decrease of the photosynthetic pigment (especially chlorophyll b) abundance in the mutant leaves, which is probably resulted from chlorophyll over degradation. To reveal the candidate genes contributing to leaf-color variation, we performed 454 transcriptome sequencing and *de novo* assembly for *Cymbidium* 'Dharma'. Roughly 0.7 million ESTs were obtained and assembled into 103,295 isotigs and 68,460 genes, representing orthologs of known plant genes as well as potential new genes. This systems bioinformatics survey, combined with molecular biology analysis, is a feasible approach to elucidate the scent of various biological processes and identify the relevant genes. Regarding the chlorophyll metabolism pathway which directly controls leaf color variation, 95 candidate genes were identified as involved. From which, we cloned 16 key enzyme-encoding genes associated with chlorophyll metabolism. After comparing the expression levels of these candidate genes, we confirmed that the expression levels of two key enzyme-encoding genes for chlorophyll degradation were greater in yellow-color mutant leaves, as was consistent with their lower chlorophyll content. Our result suggested that the mutant leaves arose probably from chlorophyll degradation and not biosynthesis. To the best of our knowledge, this study represents the first transcriptome sequencing this work represents the first study focusing on leaf varation of *Cymbidium sinense* at both physiological and molecular levels, and it supplies a further molecular basis for leaf color-associated genes. The informative EST dataset we obtained can be used as an important resource to facilitate gene discovery for molecular breeding, the identification of marketable traits, and the investigation of various biological process in *Cymbidium*.

## Supporting Information

S1 Fig Plant materials used for 454-pyrosequencing. (TIF)

S2 Fig Sequence alignment of CHLG (chlorophyll synthase) amino acids with its homologues. (TIF)

S3 Fig Sequence alignment of CLH2 (chlorophyllase) amino acids with its homologues. (TIF)

S4 Fig Phylogenetic tree of CHLG (chlorophyll synthase) in *Cymbidium sinense* 'Dharma' with its homologues in plants. (JPG)

S5 Fig Phylogenetic tree of CLH2 (chlorophyllase) in *Cymbidium sinense* 'Dharma' with its homologues in plants. (JPG)

S1 Table The primers used for qRT-PCR. (TXT)

S2 Table Results of Amplified Fragment Length Polymorphism analysis in *Cymbidium sinense* ‘Drama’ with 9 primer combinations. (DOC)

S3 Table Morphologic characteristics of *Cymbidium sinense* 'Dharma' used for Phylogenetic analysis. (DOC)

S4 Table Summary of the GOslim annotation. (TXT)

S5 Table Summary of the KEGG pathway and their corresponding gene number. (XLS)

S6 Table The isotigs involved in chlorophyll metabolism annotated by KEGG pathway. (TXT)

S7 Table Sequences of the eight identified key enzyme-encoding genes in chlorophyll metabolism pathway. (TXT)

## References

[1] Liu ZJ, Chen SC, Ru ZZ, Chen LJ, editors (2006) Chinese *Cymbidium* plants. Beijing: Science Press.

[2] Huang J, Dai S (1998) The numerical taxonomy of Chinese *Cymbidium* . Journal of Beijing Forestry University 20: 38–43.

[3] Xu Y, Teo LL, Zhou J, Kumar PP, Yu H (2006) Floral organ identity genes in the orchid *Dendrobium crumenatum* . Plant J 46: 54–68. 16553895 10.1111/j.1365-313X.2006.02669.x

[4] Aceto S, Gaudio L (2011) The MADS and the Beauty: Genes Involved in the Development of Orchid Flowers. Curr Genomics 12: 342–356. 10.2174/138920211796429754 22294877 PMC3145264

[5] Yukawa T, Stern WL (2002) Comparative vegetative anatomy and systematics of *Cymbidium* (Cymbidieae: Orchidaceae). Bot J Linn Soc 138: 383–419.

[6] Li YH, Imai K, Ohno H, Matsui S (2004) Effects of acclimatization temperatures on antioxidant enzyme activities in mericlones of a cattleya hybrid. J Jpn Soc Hortic Sci 73: 386–392.

[7] Zhang JX, Wu KL, Zeng SJ, da Silva JAT, Zhao XL, Tian CE, et al (2013) Transcriptome analysis of *Cymbidium* sinense and its application to the identification of genes associated with floral development. BMC Genomics 14. 10.1186/1471-2164-14-279 PMC3639151 23617896

[8] Chugh S, Guha S, Rao IU (2009) Micropropagation of orchids: A review on the potential of different explants. Scientia Horticulturae 122: 507–520.

[9] Leitch IJ, Kahandawala I, Suda J, Hanson L, Ingrouille MJ, Chase MW, et al (2009) Genome size diversity in orchids: consequences and evolution. Annals of Botany 104: 469–481. 10.1093/aob/mcp003 19168860 PMC2720655

[10] Fukai S, Hasegawa A, Goi M (2002) Polysomaty in *Cymbidium* . Hortscience 37: 1088–1091.

[11] Zhang X, Ye J, Guan C, Ma H (2010) Review on Recent Progress of Orchid Seeds Germination. Northern Horticulture 19: 206–209.

[12] Strickler SR, Bombarely A, Mueller LA (2012) Designing a transcriptome next-generation sequencing project for a nonmodel plant species. Am J Bot 99: 257–266. 10.3732/ajb.1100292 22268224

[13] Schulz MH, Zerbino DR, Vingron M, Birney E (2012) Oases: robust *de novo* RNA-seq assembly across the dynamic range of expression levels. Bioinformatics 28: 1086–1092. 10.1093/bioinformatics/bts094 22368243 PMC3324515

[14] Robertson G, Schein J, Chiu R, Corbett R, Field M, Jackman SD, et al (2010) *De novo* assembly and analysis of RNA-seq data. Nat Methods 7: 909–912. 10.1038/nmeth.1517 20935650

[15] Liu L, Li Y, Li S, Hu N, He Y, et al (2012) Comparison of Next-Generation Sequencing Systems. J Biomed Biotechnol 2012: 251364 10.1155/2012/251364 22829749 PMC3398667

[16] De Paolo S, Salvemini M, Gaudio L, Aceto S (2014) De Novo Transcriptome Assembly from Inflorescence of *Orchis italica*: Analysis of Coding and Non-Coding Transcripts. PLoS ONE 9: e102155 10.1371/journal.pone.0102155 25025767 PMC4099010

[17] Hsu CC, Chung YL, Chen TC, Lee YL, Kuo YT, Tsai WC, et al (2011) An overview of the *Phalaenopsis* orchid genome through BAC end sequence analysis. BMC Plant Biol 11: 3 10.1186/1471-2229-11-3 21208460 PMC3027094

[18] Hsiao YY, Tsai WC, Kuoh CS, Huang TH, Wang HC, Wu TS, et al (2006) Comparison of transcripts in *Phalaenopsis bellina* and *Phalaenopsis equestris* (Orchidaceae) flowers to deduce monoterpene biosynthesis pathway. BMC Plant Biol 6: 14 16836766 10.1186/1471-2229-6-14 PMC1540424

[19] Tsai WC, Hsiao YY, Lee SH, Tung CW, Wang DP, Wang HC, et al (2006) Expression analysis of the ESTs derived from the flower buds of *Phalaenopsis equestris* . Plant Science 170: 426–432.

[20] Xu Y, Teo LL, Zhou J, Kumar PP, Yu H (2006) Floral organ identity genes in the orchid *Dendrobium crumenatum* . Plant J 46: 54–68. 16553895 10.1111/j.1365-313X.2006.02669.x

[21] Tan J, Wang HL, Yeh KW (2005) Analysis of organ-specific, expressed genes in *Oncidium* orchid by subtractive expressed sequence tags library. Biotechnol Lett 27: 1517–1528. 16231226 10.1007/s10529-005-1468-8

[22] Teh SL, Chan WS, Abdullah JO, Namasivayam P (2011) Development of expressed sequence tag resources for *Vanda* Mimi Palmer and data mining for EST-SSR. Mol Biol Rep 38: 3903–3909. 10.1007/s11033-010-0506-3 21116862

[23] Li X, Luo J, Yan T, Xiang L, Jin F, Qin D, et al (2013) Deep sequencing-based analysis of the *Cymbidium ensifolium* floral transcriptome. PLoS ONE 8: e85480 10.1371/journal.pone.0085480 24392013 PMC3877369

[24] Su CL, Chen WC, Lee AY, Chen CY, Chang YC, Chao YT, et al (2013) A modified ABCDE model of flowering in orchids based on gene expression profiling studies of the moth orchid *Phalaenopsis aphrodite* . PLoS ONE 8: e80462 10.1371/journal.pone.0080462 24265826 PMC3827201

[25] Zhang J, Wu K, Zeng S, Teixeira da Silva JA, Zhao X, Tian CE, et al (2013) Transcriptome analysis of *Cymbidium* sinense and its application to the identification of genes associated with floral development. BMC Genomics 14: 279 10.1186/1471-2164-14-279 23617896 PMC3639151

[26] Lorenzen CJ (1966) A method for the continuous measurement of *in vivo* chlorophyll concentration. Deep Sea Research 13: 223–227.

[27] International Brachypodium Initiative. (2010) Genome sequencing and analysis of the model grass *Brachypodium distachyon* . Nature 463: 763–768. 10.1038/nature08747 20148030

[28] Conesa A, Gotz S, Garcia-Gomez JM, Terol J, Talon M & Robles M (2005) Blast2GO: a universal tool for annotation, visualization and analysis in functional genomics research. Bioinformatics 21: 3674–3676. 16081474 10.1093/bioinformatics/bti610

[29] Tatusov RL, Fedorova ND, Jackson JD, Jacobs AR, Kiryutin B, Koonin EV, et al (2003) The COG database: an updated version includes eukaryotes. BMC Bioinformatics 4: 41 12969510 10.1186/1471-2105-4-41 PMC222959

[30] Kanehisa M, Goto S (2000) KEGG: kyoto encyclopedia of genes and genomes. Nucleic Acids Res 28: 27–30. 10592173 10.1093/nar/28.1.27 PMC102409

[31] Wang XW, Luan JB, Li JM, Bao YY, Zhang CX & Liu SS. (2010) *De novo* characterization of a whitefly transcriptome and analysis of its gene expression during development. BMC Genomics 11: 400 10.1186/1471-2164-11-400 20573269 PMC2898760

[32] Zhang XM, Zhao L, Larson-Rabin Z, Li DZ, Guo ZH (2012) *De novo* sequencing and characterization of the floral transcriptome of *Dendrocalamus latiflorus* (Poaceae: Bambusoideae). PLoS ONE 7: e42082 10.1371/journal.pone.0042082 22916120 PMC3419236

[33] Hörtensteiner S, Kräutler B (2011) Chlorophyll breakdown in higher plants. BBA- Bioenergetics 1807: 977–988. 10.1016/j.bbabio.2010.12.007 21167811

[34] Shi D, Zheng X, Li L, Lin W, Xie W, Yang J, et al (2013) Chlorophyll deficiency in the maize elongated *mesocotyl2* mutant is caused by a defective heme oxygenase and delaying grana stacking. PLoS ONE 8: e80107 10.1371/journal.pone.0080107 24244620 PMC3823864

[35] Reinbothe S, Reinbothe C (1996) Regulation of chlorophyll biosynthesis in angiosperms. Plant Physiol 111: 1–7. 12226272 10.1104/pp.111.1.1 PMC157807

[36] Eckhardt U, Grimm B, Hortensteiner S (2004) Recent advances in chlorophyll biosynthesis and breakdown in higher plants. Plant Mol Biol 56: 1–14. 15604725 10.1007/s11103-004-2331-3

