# Supplementary figures and images for: Transcriptome Characterization of Cymbidium sinense 'Dharma' Using 454 Pyrosequencing and Its Application in the Identification of Genes Associated with Leaf Color Variation
Source: PLoS One. 2015 Jun 4;10(6):e0128592. doi: 10.1371/journal.pone.0128592 (PMC4456352; doi:10.1371/journal.pone.0128592)

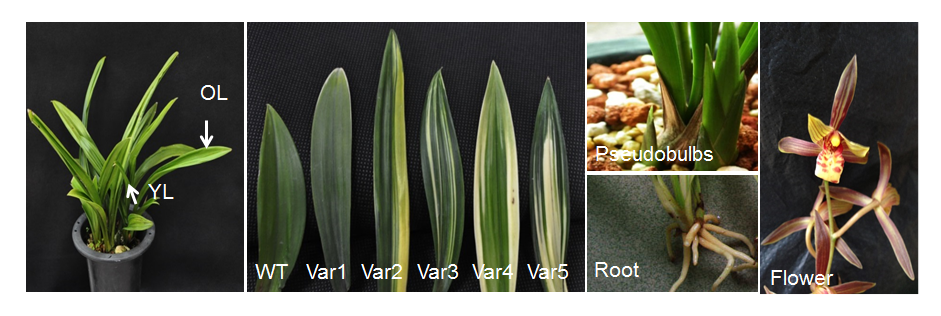

Supplement: S1 Fig — (TIF) [file pone.0128592.s001.tif]

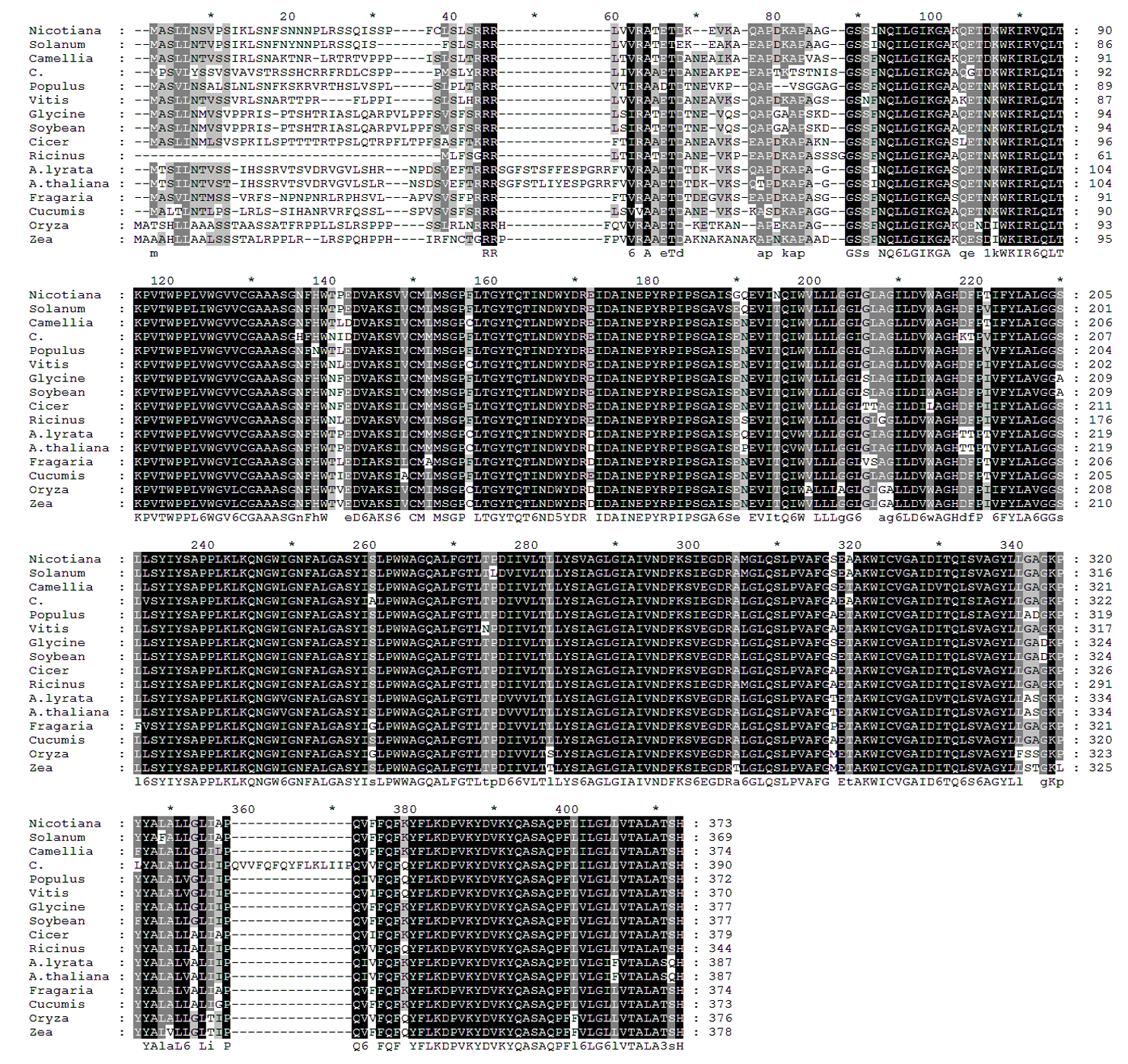

Supplement: S2 Fig — (TIF) [file pone.0128592.s002.tif]

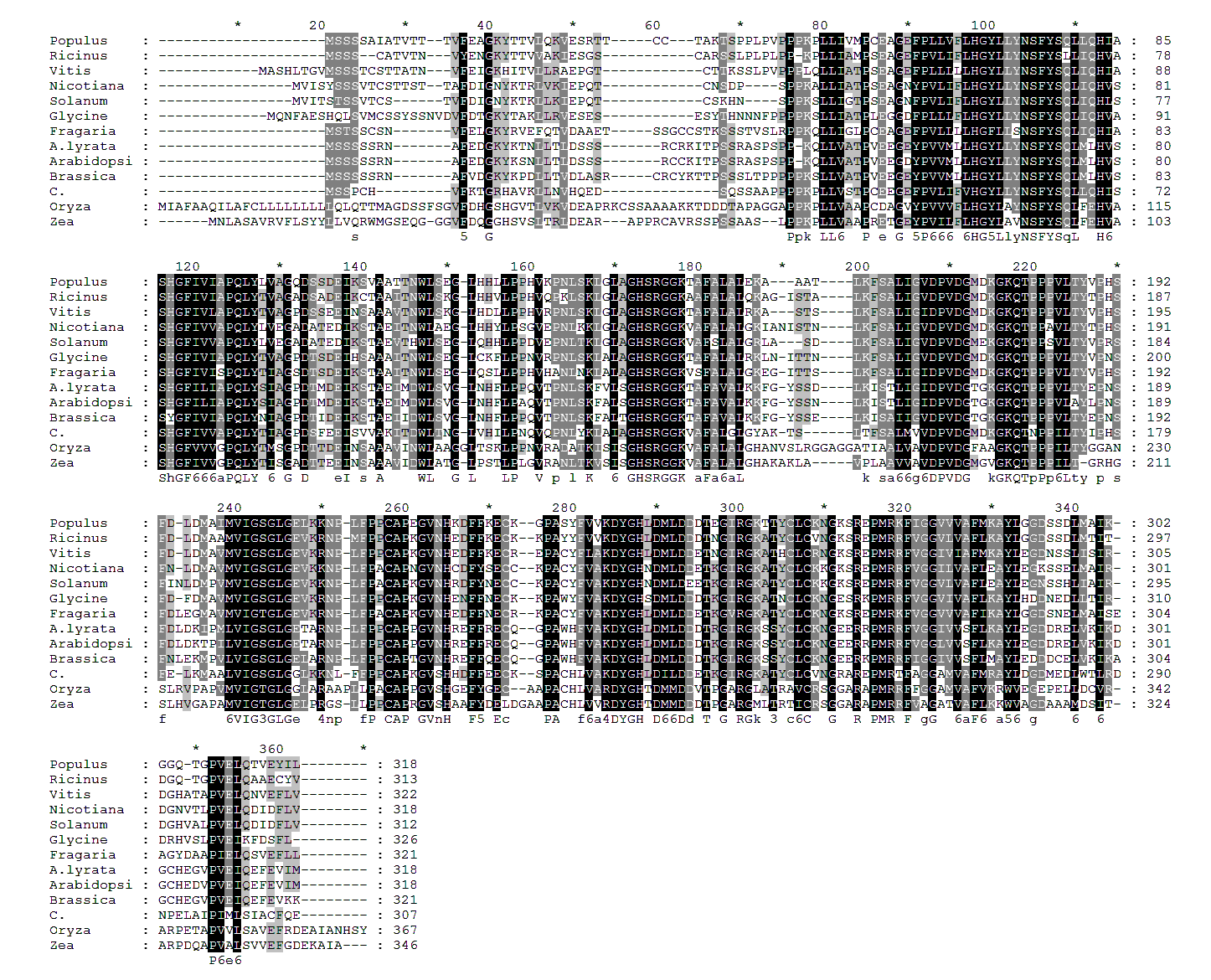

Supplement: S3 Fig — (TIF) [file pone.0128592.s003.tif]

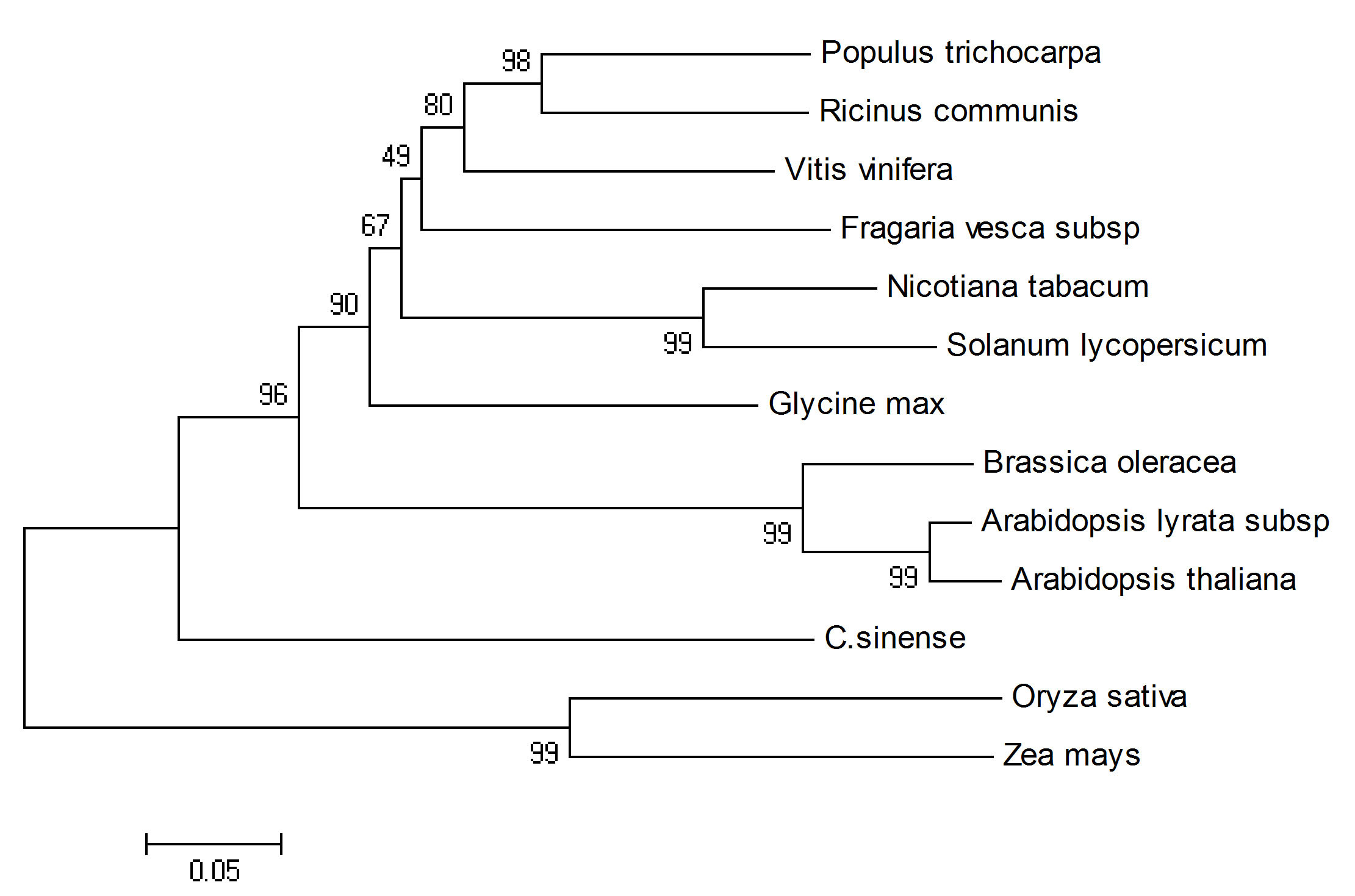

Supplement: S4 Fig — (JPG) [file pone.0128592.s004.jpg]

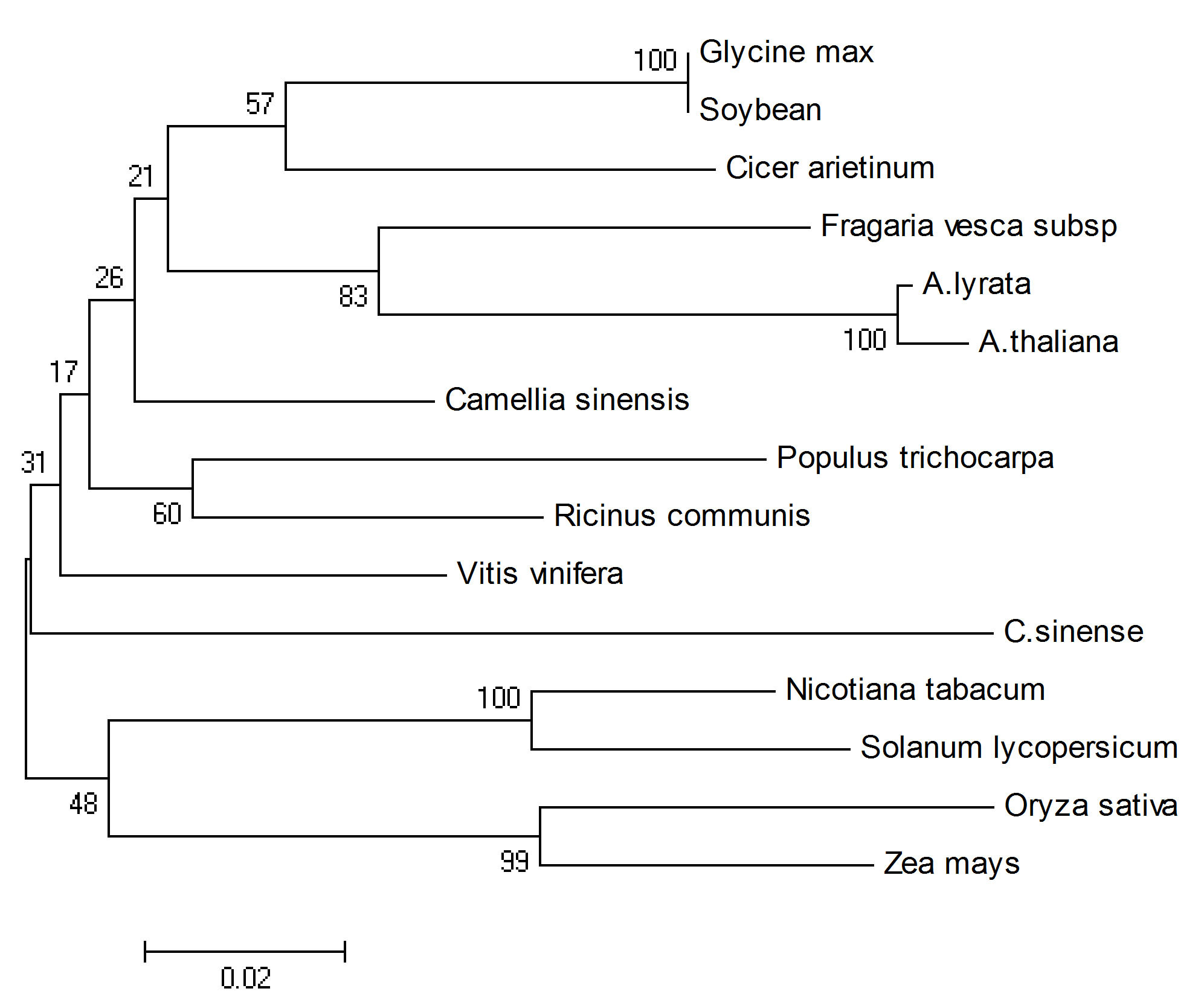

Supplement: S5 Fig — (JPG) [file pone.0128592.s005.jpg]
