# Supplementary material for: Transcriptome Characterization of Cymbidium sinense 'Dharma' Using 454 Pyrosequencing and Its Application in the Identification of Genes Associated with Leaf Color Variation
Source: PLoS One. 2015 Jun 4;10(6):e0128592. doi: 10.1371/journal.pone.0128592 (PMC4456352; doi:10.1371/journal.pone.0128592)
Supplement: S2 Table — (DOC) [file pone.0128592.s007.doc]

Table S2. Amplified results of *Cymbidium sinense* ‘Drama’ with 9 AFLP primers

| Primer pairses | Amplified bands | Polymorphic bands | Polymorphic ratio |
| --- | --- | --- | --- |
| *Pst*I GAC/ *Mse*I CTA | 72 | 71 | 98.6 |
| *Pst*I GAC/ *Mse*I CTT | 103 | 77 | 74.8 |
| *Pst*I GAG/ *Mse*I CAG | 75 | 47 | 62.7 |
| *Pst*I GAG/ *Mse*I CTC | 88 | 61 | 69.3 |
| *Pst*I GAG/ *Mse*I CTT | 73 | 56 | 76.7 |
| *Pst*I GAT/ *Mse*I CAG | 98 | 74 | 75.5 |
| *Pst*I GTG/ *Mse*I CAG | 109 | 76 | 69.7 |
| *Pst*I GTG/ *Mse*I CTG | 101 | 83 | 82.2 |
| *Pst*I GTT/ *Mse*I CAG | 127 | 103 | 81.1 |
| Mean | 94 | 72 | 76.6 |
