# Supplementary material for: Transcriptome Characterization of Cymbidium sinense 'Dharma' Using 454 Pyrosequencing and Its Application in the Identification of Genes Associated with Leaf Color Variation
Source: PLoS One. 2015 Jun 4;10(6):e0128592. doi: 10.1371/journal.pone.0128592 (PMC4456352; doi:10.1371/journal.pone.0128592)
Supplement: S3 Table — (DOC) [file pone.0128592.s008.doc]

Table S3. Morphologic characteristics of *Cymbidium sinense* ‘Dharma’ used for Phylogenetic analysis

| *Number* | *Name* | *Character* | *Orgin* | *Year* | *Leaf* | *Flower* |
| --- | --- | --- | --- | --- | --- | --- |
| Cs1 | Da Mo | Dwarf leaves | Taiwan | 1973 | Half pendulous leaf | Purple |
| Cs2 | Guan Yi Da Mo 1 | Dwarf and variegation | Taiwan | 2001 | Half pendulous leaf, Guan Yi | Purple |
| Cs3 | Guan Yi Da Mo 2 | Dwarf and variegation leaves | Guanag Dong | 2003 | Half pendulous leaf, Guan Yi | Purple |
| Cs4 | Guan Yi Da Mo 3 | Dwarf and variegation leaves | Guanag Dong | 2003 | Half pendulous leaf, GuanYi | Purple |
| Cs5 | Zhua Yi Da Mo 1 | Dwarf and variegation leaves | Taiwan | 2001 | Half pendulous leaf, Zhua Yi | Purple |
| Cs6 | Gao Yi Da Mo 1 | Dwarf and variegation leaves | Taiwan | 2001 | Half pendulous leaf, Gao Yi | Purple |
| Cs7 | He Yi Da Mo | Dwarf and variegation leaves | Taiwan | 2001 | Half pendulous leaf, He Yi | Purple |
| Cs8 | Ban Gao Yi Da Mo | Dwarf and variegation leaves | Guanag Dong | 2001 | Half pendulous leaf, Ban Gao Yi | Purple |
| Cs9 | Gao Yi Da Mo 2 | Dwarf and variegation leaves | Taiwan | 2001 | Half pendulous leaf, Gao Yi | Purple |
| Cs10 | Da Mo 2 | Dwarf leaves | Guanag Dong | 2002 | Half pendulous leaf | Dark Purple |
